# Pro-inflammatory markers in patients with obstructive sleep apnea and the effect of Continuous Positive Airway Pressure therapy

**DOI:** 10.5935/1984-0063.20200117

**Published:** 2022

**Authors:** Jamil Al-Mughales, Siraj Omar Wali, Md. Dilshad Manzar, Faris Alhejaili, David Gozal

**Affiliations:** 1 King Abdulaziz University Hospital, Diagnostic Immunology Division, Department of Clinical Laboratory Medicine - Jeddah - Jeddah - Saudi Arabia.; 2 Faculty of Medicine, King Abdulaziz University, Department of Medical Microbiology and Parasitology Immunology Division - Jeddah - Jeddah - Saudi Arabia.; 3 King Abdulaziz University, Sleep Medicine and Research Center, Sleep Medicine Research Group, Internal Medicine Department, Faculty of Medicine - Jeddah - Jeddah - Saudi Arabia.; 4 Majmaah University, Department of Nursing, College of Applied Medical Sciences - Majmaah - Majmaah - Saudi Arabia.; 5 The University of Missouri School of Medicine, Department of Child Health and the Child Health Research Institute - Columbia - Columbia - United States.

**Keywords:** Inflammation, Sleep Apnea Syndromes, C-Reactive Protein, Fibrinogen

## Abstract

**Objectives:**

To evaluate the association of obstructive sleep apnea (OSA) with high-sensitivity C-reactive protein (CRP) and fibrinogen levels and to assess the effect of short-term therapy using continuous positive airway pressure (CPAP).

**Material and Methods:**

A prospective, open-label, controlled trial was conducted among clinically referred patients at risk for OSA undergoing diagnostic polysomnography (PSG). After PSG, the patients were divided into 3 groups: OSA treatment group (TG) (n=21), untreated OSA group (UOG) (n=19), and non-OSA healthy control group (HCG) (n=24). CRP and fibrinogen levels were measured at baseline and one month after treatment. Repeated-measures (RM) ANOVA and ANCOVA were used to compare changes in CRP and fibrinogen levels among the three groups by analyzing between-subject and within-subject effects as functions of time and adjusting for significant covariates.

**Results:**

At baseline, OSA subjects had significantly higher CRP [t(52.37)=-2.46, p=0.02)] and fibrinogen levels [t(57)=-2.00, p=0.05)] than HCG subjects. No significant differences in CRP levels [(F(2,58)=2.29, p=0.11)] or fibrinogen levels [(F(2, 58)=1.28, p=0.29)] emerged between TG and HCG subjects after adjusting for the pretest levels.

**Conclusion:**

CPAP therapy for one month does not affect CRP and fibrinogen levels among moderate-to-severe OSA patients. However, OSA is associated with elevated levels of these inflammatory biomarkers.

## INTRODUCTION

Obstructive sleep apnea (OSA) is a common sleep-related breathing disorder that is characterized by repeated complete or partial collapse of the upper airway during sleep^1,2,3^. OSA results in momentary intermittent hypoxemia and hypercapnia, sleep fragmentation, and poor sleep quality^4^. OSA has clear negative impacts on the quality of life of affected individuals. This condition is also associated with adverse safety and health consequences, including cardiovascular and cerebrovascular diseases, type 2 diabetes, cognitive impairments, depression, and ocular conditions^5,6^. Moreover, OSA is a risk factor for cardiovascular disease, probably due to increases in systemic inflammation and oxidative stress and their injurious effects on the vascular endothelium^7^. This status of increased systemic inflammation may result in initiation and acceleration of underlying atherosclerosis with consequent increases in morbidity and mortality^8,9^. Indeed, the recent literature supports a conceptual framework wherein OSA should be considered a low-grade chronic systemic inflammatory disorder^10^.

Previous studies proposed that OSA modulates the expression and secretion of inflammatory markers from fat and other tissues^10,11^. In fact, independent of obesity, elevated levels of proinflammatory factors, including C-reactive protein (CRP), fibrinogen, tumor necrosis factor-α and interleukin-6, have been reported among patients with OSA^10^. Hence, ongoing inflammatory responses have been suggested to play important roles in the association between OSA and chronic inflammation-induced pathologies, such as atherosclerosis^12^. The activation of inflammatory responses through adaptive pathways in OSA may be an important molecular mechanism underlying the development of cardiovascular disease and a variety of other metabolic diseases^13,14^.

Continuous positive airway pressure (CPAP) remains the most effective therapy to date for improving the polysomnography-derived parameters indicating OSA severity, i.e., the apnea hypopnea index (AHI), oxygen desaturation index 3% (ODI3) and nadir oxyhemoglobin saturation (SaO_2_)^4,15,16^. Thus, exploring the potential relationships between systemic inflammatory markers in OSA and the effect of CPAP-based treatment is important. There are conficting data in the literature regarding the efficacy of CPAP in reducing the levels of proinflammatory biomarkers and, consequently, providing an overall protective effect against the development of atherosclerosis and cardiovascular diseases^17,18,19,20,21^. This study was conducted to explore the effect of short-term CPAP therapy on the levels of two well-established proinflammatory biomarkers, namely, CRP and fibrinogen, which are regarded as biological indicators of ongoing systemic inflammation.

## MATERIAL AND METHODS

### Design and setting

This was a prospective controlled trial that was conducted from April 2018 to May 2019. The trial took place at the Sleep Medicine and Research Center (SMRC) at King Abdul-Aziz University Hospital (KAUH), Jeddah, Saudi Arabia.

### Population

All patients with a clinical suspicion of OSA who were referred to the SMRC for diagnostic polysomnography (PSG) were included in the study. The exclusion criteria included a past diagnosis of OSA, treatment for OSA, presence of conditions that could affect the levels of inflammatory markers, including history of respiratory and cardiovascular diseases with chronic hypoxia, neuromuscular disorders, infectious diseases, rheumatological diseases, immunological diseases, tumors, peripheral vascular disease, liver or kidney diseases, coagulopathy, psychogenic disorders, and diabetes mellitus; history of trauma or surgery in the past 3 months; recent use (past 3 months) of corticosteroids, antibiotics, immune suppressors, or hormones; and smoking history in the past 6 months.

### Procedures

#### Initial assessment

All patients referred to the SMRC for a sleep study underwent data collection and physical examination. Demographic data, including age, sex, body mass index (BMI), neck circumference, and blood pressure, were collected. The Epworth sleepiness scale (ESS) was used to assess daytime sleepiness^22^. All eligible subjects were invited to participate and signed an informed consent form that was approved by the local institutional review board (IRB) (reference number 178-18).

### Overnight PSG

All participants underwent overnight PSG using a standard montage, as previously described^23^. The sleep stages and respiratory events were scored according to the guidelines of the American Academy of Sleep Medicine (AASM)^24^. The severity of OSA was determined according to the AASM recommendations using the AHI: 5-14, mild; 15-29, moderate; and ≥30, severe^25^.

### Group allocation

**Figure 1.**
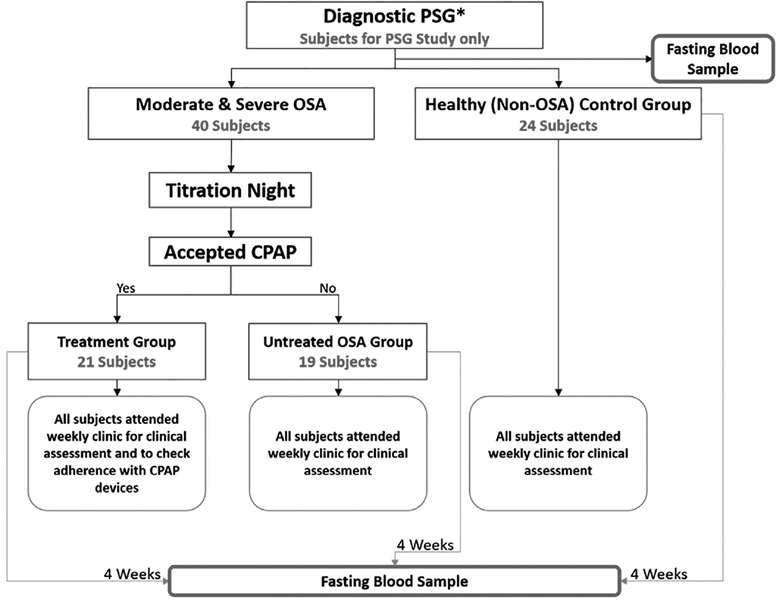
Flowchart of the study procedure.

According to the PSG results, participants were categorized into 3 groups:

The first group included those with moderate-to- severe OSA according to the AASM definition^25^, i.e., those with an AHI of 15/hour of total sleep time (hrTST) or greater, who accepted initiation treatment with CPAP (treatment group [TG]). The second group included patients with moderate-to-severe OSA who refused CPAP treatment (untreated OSA group [UOG]). The third group included participants without OSA, i.e., those with an AHI less than 5 (healthy control group [HCG]) (Figure 1).

### Initial proinflammatory marker measurements

Fasting blood samples were collected from all study participants to measure the serum and plasma levels of proinflammatory markers in the morning after the diagnostic PSG. Venous blood samples were collected into 5ml tubes with and without added anticoagulation factors, and the serum and plasma were separated and stored at -80°C immediately.

Commercially available assays were used to quantify high-sensitivity CRP (Siemens; Germany) and fibrinogen (ACL TOP 550 CTS Instrumentation Laboratory, Italy) levels according to the manufacturer’s instructions. The CRP and fibrinogen assays exhibited analytical sensitivities less than 3.4mg/l and 50mg/dl, respectively. Serum CRP concentrations were measured using fully automated BNII nephelometer (Siemens; Germany). The cutoff value for a normal CRP level was 3.4mg/l. Fibrinogen was measured in citrated plasma by an ACL TOP 550 instrument (Instrumentation Laboratory, Italy). The measurement range was between 180 and 350mg/dl. Plasma heparin concentrations below 2U/ml did not affect the test. Concentrations of CRP>3.4mg/l and fibrinogen >350mg/dl were considered elevated.

## CPAP THERAPY

All participants with OSA who agreed to initiate treatment underwent a CPAP titration PSG study to determine the optimal CPAP pressure according to the AASM guidelines^19^, and this pressure was then implemented at home.

### Follow-up and endpoints

The three groups were followed for one month following the diagnostic PSG. Follow-up included weekly visits to determine whether there were any necessary changes to the medications or events that could affect the levels of the inflammatory markers and to ensure adherence to CPAP treatment in the TG using data from memory cards installed within the CPAP devices^26^. Adherence to CPAP was considered acceptable when the minimum CPAP use was ≥4 hours/70% of the nights. At the end of the one-month follow-up, fasting morning blood samples were drawn to analyze the levels of the proinflammatory biomarkers, which were processed as described above. Participants who did not meet the adherence criteria were excluded.

### Statistical methods

All of the data analyses in this study were performed using the Statistical Package for Social Sciences version 26.0 for Windows (SPSS Inc., Chicago, IL, USA). Means, standard deviations, frequencies and percentages were used to describe the data. Chi-square tests, maximum likelihood ratio chi-square tests, and one-way and Kruskal-Wallis tests were used to show differences in the participants’ characteristics. CRP and fibrinogen levels were not normally distributed and were therefore log-transformed for further analysis. One-way ANOVA and one-way ANCOVA were used to assess differences in the log-transformed CRP and log-transformed fibrinogen values. Principal component analysis was used to estimate a single common component (age, BMI, neck circumference, AHI, and sex) to use as a covariate to adjust one-way ANCOVA. Binary logistic regression was used to assess association between OSA, inflammatory markers, and other factors. The model was explored with OSA condition (yes/no) as dependent variable and variables such as sex, age, BMI, neck circumference, systolic blood pressure (BP), diastolic B P, ESS score, STOP-BANG classification, log-transformed CRP, and log-transformed fibrinogen as independent variables. A two-tailed *p*-value<0.05 was considered statistically significant.

## RESULTS

### Participant characteristics

The baseline characteristics of the study participants are shown in Table 1. There was no significant difference in sex distribution among the three groups (p=0.26); however, TG subjects were significantly older (51.9±12.9 years) than UOG (43.1±12.5 years) and HCG (29.6±8.8 years) subjects (p<0.01). Additionally, BMI (p=0.04) and neck circumference (p<0.01) differed significantly among the three groups, whereas ESS score (p=0.52) and BP (systolic: p=0.27; diastolic: p=0.12) were similar among groups. There were no statistically significant differences among the three groups in log-transformed CRP values (F(2,58)=2.45, p=0.10) or log-transformed fibrinogen values (F(2,58)=1.99, p=0.14).

**Table 1.** Characteristics of the subjects in the three groups.

Characteristics	HCG	TG	UOG	P value
Gender				
Female	12 (60.0%)	7 (35.0%)	8 (42.1%)	.263
Male	8 (40.0%)	13 (65.0%)	11 (57.9%)	
Age	29.6±8.77	51.95±12.89	43.10±12.51	<.01
BMI	30.40±9.43	36.16±12.08	39.35±11.02	.04
Neck circumference	34.18±5.91	38.24±4.51	40.16±3.26	.001
Blood pressure				
Systolic	131.7±16.62	134.00±14.26	142.24±28.32	.270
Diastolic	72.45±7.82	78.68±18.33	86.24±29.46	.122
STOP-BANG				
Low risk	13 (65.0%)	4 (20.0%)	5 (26.3%)	.
Intermediate risk	2 (10.0%)	7 (35.0%)	3 (15.8%)	018
High risk	5 (25.0%)	9 (45.0%)	11 (57.9%)	
ESS	13.60±6.25	11.40±6.18	12.16±6.06	.523
AHI	2.52±1.85	40.78±26.80	32.98±20.25	<.01

### Baseline differences in fibrinogen and CRP levels among OSA participants

At baseline, participants with OSA had significantly higher baseline serum log-transformed CRP levels (0.80±0.35mg/dl) than HCG subjects (0.61±0.24mg/dl; actual CRP values: 6.35±2.24mg/ dl versus 4.10±1.73mg/dl, t(52.37)=-2.46, *p*=0.02). Similarly, participants with OSA had significantly higher baseline serum log-transformed fibrinogen levels (2.51±0.12mg/dl) than HCG subjects (2.45±0.12mg/dl; actual fibrinogen levels: 323.59±1.32mg/dl versus 281.84±1.32mg/dl, t(57)=-2.00, *p*=0.05).

### Baseline differences between the OSA groups: age, BMI, AHI, neck circumference, and STOP-BANG classification

Age was significantly higher in the TG (51.95±12.89 years) than in the UOG (43.10±12.51 years, t(37)=2.17, *p*=0.04) (Table 2).

**Table 2.** Baseline differences in age and AHI between OSA groups.

Characteristics	Mean ± SD/number (percentage)		p-value
	TG (n=21)	UOG (n=19)	
**Age (years)**	51.95 ± 12.89	43.10 ± 12.51	0.036
**AHI**	40.78 ± 26.80	32.98 ± 20.25	0.31
**BMI**	36.1 6 ± 12.08	39.35 ± 11.02	0.395
**Neck circumference**	38.24 ± 4.51	40.16 ± 3.27	0.139
**STOP-BANG**			
Low risk	4 (20.0%)	5 (26.3%)	
Intermediate risk	7 (35.0%)	3 (15.8%)	0.381
High risk	9 (45.0%)	11 (57.9%)	

### Baseline differences in fibrinogen and CRP levels between the treated and untreated OSA groups

There were no significant differences in the log-transformed CRP levels (F(1, 39)=0.17, *p*=0.68) and log-transformed fibrinogen levels (F(1,39)=2.45, *p*=0.86) between the TG and UOG groups at baseline, even after adjusting for covariates, including age, BMI, neck circumference, and AHI (log-transformed CRP: (F(1,39)=0.16, *p*=0.69; log-transformed fibrinogen: F(1,39)=0.02, *p*=0.88) (Table 3).

**Table 3.** Fibrinogen and CRP levels in the treated and nontreated OSA groups.

Serum inflammatory marker level	Mean ±SD		p-value (unadjusted)	p-value (adjusted)
	TG (n=21)	UOG (n=19)		
Log-transformed CRP (mg/dl)	0.78 ± 0.37	0.83 ± 0.34	0.679	0.689
Log-transformed fibrinogen (mg/dl)	2.51 ± 0.13	2.52 ± 0.12	0.863	0.875

A common component score that could be used as a covariate in the model was generated using principal component analysis for age, BMI, neck circumference, AHI, and sex. This common component score explained 34.21% of the variability in age, BMI, neck circumference, AHI and sex. The homogeneity of the regression condition was determined to be satisfactory, with a non-significant interaction between groups (F(1,39)=2.61; *p*=0.12) and log-transformed CRP as the dependent variable. Similarly, there was no significant interaction between groups (F(1,39)=1.76; *p*=0.19), with log-transformed fibrinogen as the dependent variable.

### Association of inflammatory markers and other variables with OSA at baseline

The binary logistic regression model for OSA was statistically significant when compared to the model with only intercepts; χ^2^(11, N=59)=43.44, *p*<0.01. The prediction model with 10 factors accounted for 74.1% (Nagelkerke R Square) of the variance in the classification of OSA. The model correctly classifed 89.3% of the cases (sensitivity=94.4% and specificity=80.0%), with increasing age being associated with an increasing likelihood of OSA (adjusted odds ratio (AOR)=1.26, 95% confidence interval (CI): 1.07-1.48; *p*<0.01).

### CRP levels: within-group pre-post difference

**Figure 2.**
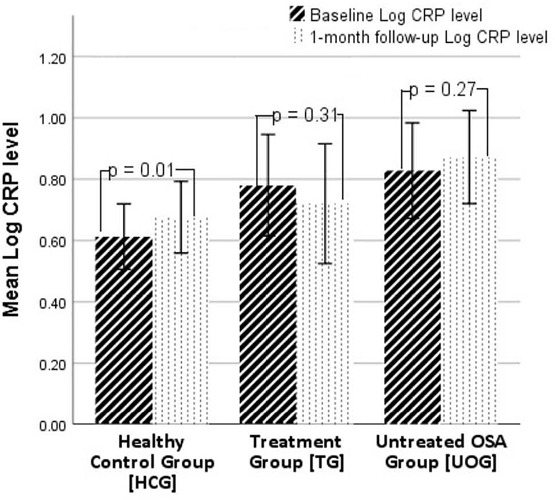
Mean CRP levels at baseline and at 1-month follow-up in the HCG and the two OSA groups. TG: OSA Treatment Group, UOG: Untreated OSA Group, HCG: Healthy Control Group.

At the 1-month follow-up, the log-transformed CRP levels were significantly higher in the HCG group than in the baseline group (0.68±0.26mg/dl vs. 0.61±0.24mg/dl; t(19)=-3.07, *p*=0.01). In addition, CPAP treatment was not associated with any statistically significant differences in CRP levels (Figure 2).

### Fibrinogen levels: within-group pre-post difference

**Figure 3.**
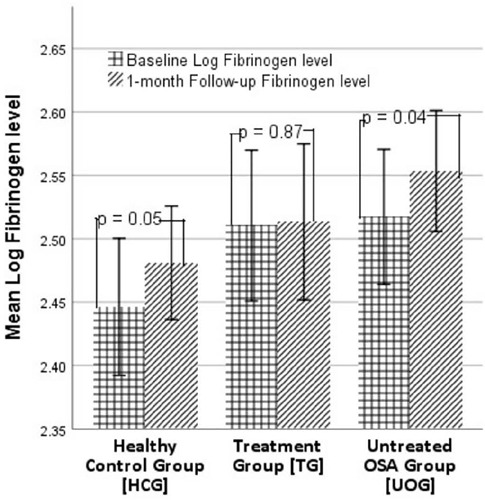
Mean fibrinogen levels at baseline and at 1-month follow-up in the HCG, TG and UOG groups. TG: OSA Treatment Group, UOG: Untreated OSA Group, HCG: Healthy Control Group.

At the 1-month follow-up, the fibrinogen levels were significantly increased in the HCG compared with that at baseline (2.48±0.10mg/dl vs. 2.45±0.12mg/dl at baseline; t(19)=-2.13, *p*=0.05) (Figure 2). No significant changes after CPAP treatment emerged in fibrinogen level in the TG subjects (*p*=0.87), and similar to the HCG, the UOG also exhibited significant increases in fibrinogen levels at the 1-month follow-up (2.55±0.10mg/dl vs. baseline: 2.52±0.12mg/dl; t(18)=-2.17, *p*=0.04) (Figure 3).

### Effect of 1 month of CPAP therapy on CRP and fibrinogen levels

ANCOVA revealed that there were no significant differences in the log-transformed fibrinogen levels between the treated group and the untreated groups after 1 month of CPAP therapy after adjusting for baseline levels (F(2,58)=1.28, *p*=0.29), and similar findings emerged for the log-transformed CRP levels (F(2,58)=2.29, *p*=0.11).

## DISCUSSION

Increased levels of CRP and fibrinogen are detectable among OSA patients, supporting the conceptual framework that OSA is a chronic low-grade systemic inflammatory condition. However, adherence to CPAP treatment for one month did not lead to significant changes in these inflammatory markers. Nevertheless, while a significant increase in the fibrinogen level after 4 weeks of follow-up was observed in both the HCG and untreated OSA group compared to baseline, no such increases were detectable in the CPAP-treated subjects, suggesting that rather than reversing the expression of inflammatory biomarkers, CPAP treatment would be expected to abrogate the temporal progression of inflammation.

The current study supports previous published findings indicating that OSA is a chronic inflammatory condition^10,14^. Nadeem et al. (2013)^10^ conducted a meta-analysis that included 51 studies and concluded that the levels of inflammatory markers are higher in patients with OSA than in control subjects. Furthermore, Li et al. (2017)^14^, in another meta-analysis, reported the link between serum CRP levels and OSA and the interactive effects of obesity and the severity of OSA on CRP concentrations. More recently, however, a Korean study involving 1,835 subjects showed that CRP levels were elevated in patients with moderate and severe OSA, independent of other confounders, including obesity^27^. It is worth mentioning that although the severity of OSA is usually classifed by the AHI according to AASM^25^, however, other parameters may also determine OSA severity, such as oxygen saturation^28^. Accordingly, CRP levels was reported to correlate significantly not only with AHI but also with oxygen saturation during sleep^29^. In addition, Kim et al. (2016)^27^ reported that CRP levels were negatively associated with the SaO_2_ nadir. Nevertheless, despite the presence of elevated levels of inflammatory markers, including CRP and fibrinogen, among OSA patients, the association between the levels of inflammatory markers and OSA severity has proven elusive and inconsistent^30,31,32,33^. The tenacious investigation of these biomarkers in the context of OSA is obviously explained by the fact that these markers are strongly associated with cardiovascular risk and endothelial dysfunction, as well as with metabolic syndrome and stroke^34,35,36^.

In the present study, short-term treatment consisting of one month of adherence to CPAP therapy did not lead to obvious reductions in the levels of these inflammatory markers. Several inflammatory markers that are believed to contribute to the pathogenesis of endothelial dysfunction have been previously studied among OSA patients before and after CPAP therapy^18,37^. In line with our findings, a controlled trial tested the effect of CPAP therapy on fibrinogen levels and erythrocyte sedimentation rate (ESR) in patients diagnosed with OSA and cardiac arrhythmias and found no significant differences after 3 and 6 months of treatment compared to the corresponding values in patients who received only phar macological treatment^38^. The authors concluded that fibrinogen and ESR may not be reliable markers of the efficacy of CPAP therapy^38^. Similarly, a cohort study measured the post-CPAP therapy changes in both fibrinogen and CRP levels in patients with OSA, including those with ischemic heart disease (IHD), and found no differences in the two markers after 3 months of CPAP therapy^39^. However, when analyzing the effect of CPAP treatment on OSA patients without the clinical manifestations of IHD, a statistical trend towards a decrease in mean CRP levels was observed (*p*=0.05). These findings may suggest that a more beneficial effect of CPAP therapy should be expected among OSA patients without clinically apparent IHD^39^. We should point out that evidence for the reversibility of vascular inflammation after long-term exposure to intermittent hypoxia mimicking sleep apnea was not apparent in a mouse model and was likely explained by epigenetic changes in macrophage pathways underlying sustained inflammatory processes^40,41^.

Conversely, a meta-analysis that included nearly 1,200 OSA patients from 14 cohort studies found standardized mean differences of 0.68 and 0.74 units in CRP levels after 3 and 6 months of CPAP therapy, respectively, compared to the pre-CPAP therapy measurements^19^. The authors concluded that CRP was a reliable indicator of the efficacy of CPAP therapy and that the use of CRP concentrations could assist in the prediction of cardiovascular risk in OSA patients^19^. Another research team investigated the effect of 6 months of nasal CPAP therapy on CRP levels in patients with overlap syndrome, i.e., the coexistence of OSA with chronic obstructive pulmonary disease (COPD). The findings showed a nearly 50% decrease in the mean CRP levels from baseline, and the decline in CRP levels was linearly correlated with the number of hours of CPAP therapy utilized per night^42^. These findings concur with those of a similar trial that tested the effect of CPAP therapy on systemic inflammatory markers in patients with overlap syndrome and in those with OSA alone^43^. Nural et al. (2013)^43^ reported significant CPAP-induced decreases in CRP levels in both the overlap syndrome and OSA groups (*p*=0.04 and *p*=0.02, respectively). Sex-specific effects of CPAP therapy on CRP levels have also been postulated, whereby a better effect on CRP level may be present among males receiving 3 months of CPAP treatment, as compared to the unaltered CRP levels among females after 3 months of the same treatment. Of note, female patients required 6 months of CPAP therapy to manifest declines in CRP concentrations^44^. The discrepancy between the results of these studies and our current findings could be related to the shorter duration of CPAP in our study or could be ascribed to differences among the participant cohorts, since we excluded patients with factors that could have exacerbated their systemic inflammatory status. Nevertheless, these observations suggest that a favorable effect of CPAP may occur in terms of fibrinogen levels, as well as CRP concentrations, provided that the treatment is adhered to for a much longer period of time. More importantly, we surmise that the beneficial effects of CPAP therapy may be more apparent when illustrated by CRP levels in specific patient subgroups (i.e., men with relatively severe OSA and without clinically established cardiovascular conditions).

Similar to that of other studies, the purpose of the present study was to determine the therapeutic effect of CPAP therapy on systemic inflammation, with the ultimate goal of reducing the global cardiovascular risk among OSA patients. However, the only favorable finding supporting such assumption resides in the indirect effect of CPAP therapy, whereby treatment was accompanied by the absence of an increase in fibrinogen and CRP levels in the treated group. Notwithstanding, we cannot rule out that such changes may also be due to a cyclic or seasonal variation in inflammation biomarkers that is prevented by CPAP therapy, since notable seasonal variations were reported in a large population-based study of inflammatory biomarkers^45^.

There are several aspects and limitations of this study that should be highlighted. First, the one-month duration of CPAP therapy may not have been sufficient to induce a significant change in the plasma markers of inflammation. Second, the small sample size of the study groups may have hampered the detection of significant differences due to type 2 error. Third, due to the absence of randomization, notable selection bias may have occurred during group allocation, in which older patients and those with more severe OSA were more likely to be included in the CPAP treatment group. Fourth, patients with a CPAP use of ≥4 hours/70% of the nights was considered acceptable and included in the study. However, this adherence variable was not extracted further as level of adherence. Therefore, relationship between OSA and short-term CPAP could not be reported after adjusting for adherence level. Future studies may be better placed to record adherence level of CPAP, severity of OSA in groups, etc. All of these factors may have resulted in a reduction in the relative effect of CPAP therapy.

## CONCLUSION

CPAP therapy for one month does not affect CRP and fibrinogen levels among moderate-to-severe OSA patients. However, one month of adherence to CPAP therapy may have a favorable impact on CRP and fibrinogen levels in moderate-to-severe OSA patients by preventing temporal increases in such markers. Furthermore, our study confirms that OSA is associated with elevated levels of such inflammatory biomarkers.
